# Supplementary material for: Navigating the cholera elimination roadmap in Zambia – A scoping review (2013–2024)
Source: PLoS Negl Trop Dis. 2025 Jun 23;19(6):e0012422. doi: 10.1371/journal.pntd.0012422 (PMC12208409; doi:10.1371/journal.pntd.0012422)
Supplement: Attachment — Submitted filename: Response to Reviewers PNTD-D-24-01118-R1 Mbewe et al.pdf [file pntd.0012422.s004.pdf]

Dear Reviewers,

Thank you very much for your kind words and thoughtful feedback on our manuscript. We appreciate your detailed comments, which have helped us strengthen the clarity and relevance of our work. Please find our responses to your suggestions below:

Abstract:

Line 26: Kindly add types of study
Reply: Thank you for the suggestion. We have revised the sentence to include 'relevant qualitative and quantitative research' to clarify the types of studies included.

Line 29-31: Adding how the risk factors affect the Zambia's cholera elimination program would be beneficial.
Reply: We have rephrased this section, reading now: 'Risk factors for recurrent outbreaks, including poor access to water, sanitation, and hygiene services in unplanned urban settlements and rural fishing villages, continue to derail elimination efforts.' to better reflect their impact on national progress.

Line 34: Indicate whether high-risk areas or the entire country will host climate-resilient WASH infrastructures.
Reply: We have clarified this point by specifying that climate-resilient WASH infrastructure will be implemented "in cholera hotspots", aligning with national targeting strategies

Line 45: Keyword GTFCC – Use full form
Reply: Thank you for catching that. We have updated the keyword to read 'Global Task Force on Cholera Control (GTFCC)' in full.

Introduction:

The introduction demonstrates well about the context.

Line 66-68: Need to add cholera epidemiological data, especially for sub-Saharan Africa as well as Zambia
Reply: We have included updated global epidemiological data from WHO, noting that 'In 2024, a cumulative total of 804,721 cases and 5,805 deaths were reported across all five WHO regions in 33 countries.' The specific situation in Zambia has also been updated accordingly.

Line 76-78: Add the measures already implemented in Zambia to address the nation's effort before the 2024 outbreak for more clarity.

<u>Reply:</u> Thank you for this helpful suggestion. We have added information on national efforts, including the launch of the first Multisectoral Cholera Elimination Plan (MCEP) in 2018 and a successful pre-emptive Oral Cholera Vaccination (OCV) campaign in 2021 targeting over five million people in hotspot areas.

Line 85-86: Mentioning Healthcare system strengthening with Community engagement will add more depth. Also, use only 'WASH' in the last for better clarity.
<u>Reply:</u> We have revised the sentence to highlight the importance of both health system strengthening and community engagement and replaced the longer phrasing with 'WASH' for clarity and consistency.

Line 92-93: How one heath approach will help Zambia cholera control approach. You can add some on this.
<u>Reply:</u> Thank you for this point. We have revised the text to better reflect this linkage by stating: 'This scoping review was undertaken to summarise existing evidence on cholera epidemiology and elimination in Zambia, with particular attention to multisectoral and One Health approaches, incorporating evidence from the human-environment interface.'

Method:

Method is clear and sound.
<u>Reply:</u> Thank you kindly.

Line 119-122 Need to add some examples of research gap.
<u>Reply:</u> We appreciate the suggestion and have now included specific examples of research gaps, such as limited documentation of effective community-level interventions and insufficient evaluation of long-term WASH infrastructure sustainability in rural areas.

Results:

Line 114: Google scholar search found 11300 articles which is too high. How could you find the relevant one? Please explain and add in the section.
<u>Reply:</u> Thank you for pointing this out. The sentence has been updated to accurately reflect the number of records retrieved from Google Scholar (13 and not 11300), based on the search terms using 'cholera' AND 'Zambia', limited to January 2013 to June 2024. The corresponding numbers throughout the manuscript have been revised for consistency.

Line 151-153: A brief explanation of the impact of PRISMA rules on data extraction quality is needed for Unfamiliar readers.
<u>Reply:</u> Thank you for this suggestion. We have added a brief explanation to clarify that adherence to the PRISMA-ScR guidelines ensured a systematic and transparent approach to

study selection and data extraction, thereby enhancing the reliability, reproducibility, and comprehensiveness of the review process.

Line 165-168: Epidemiology trends over time can be clarified by additional epidemic case numbers, regions, and age groups of affected individuals.
Reply: We appreciate this helpful feedback and have now included additional data to illustrate temporal trends, highlighting the most affected province and available information on age-specific disease burden where reported, to provide a clearer picture of the evolving epidemiological profile. The revised text reads "From 2000 to 2010, 39,285 cases in total over the ten10 years, with 80% of these cases occurring in Lusaka, the capital."

Line 253-258: Details and extent of AMR is needed for better description
Reply: Thank you for your insightful comment. We have now expanded the description to include details on the extent of antimicrobial resistance (AMR) observed in cholera outbreaks. Specifically, we highlight that earlier studies reported low-level resistance to tetracyclines, while subsequent outbreaks documented resistance levels as high as 95%, likely due to its frequent use as a first-line treatment

Line 331-335: How el nino make impact on cholera outbreak? Kindly describe.
Reply: Thank you for your valuable comment. We have now elaborated on the impact of El Niño on cholera outbreaks. "El Niño events are associated with changes in rainfall patterns and temperature, which can contribute to cholera transmission. Increased rainfall may lead to flooding and contamination of water sources, while warmer temperatures can promote the growth and persistence of *Vibrio cholerae* in the environment. These conditions together create a conducive setting for the spread of cholera in vulnerable communities." The revised text now reflects this clarification.

Discussion:

Well written.

Line 390-391: Could you please add the period or time
Reply: Thank you for pointing this out. We have now added the relevant time period to clarify the context in the sentence. This addition provides better temporal framing for the reader and improves the overall clarity of the statement.

Line 435-452: Add some detailed information about the mixed results of microbiome.
Reply: Thank you for this important suggestion. We have now expanded the section to include more detail on the mixed findings from microbiome studies related to cholera transmission. "Some studies suggest that certain gut microbiota compositions may offer protective effects by inhibiting *Vibrio cholerae* colonisation, while others indicate that disruptions to the

microbiome, due to malnutrition, antibiotic use, or environmental factors, may increase susceptibility to infection. These contrasting results highlight the complex and context-dependent role of the microbiome in cholera dynamics, which warrants further investigation."

Line 418-426: You can add some causes of misconception.
Reply: Thank you for this valuable suggestion. We have revised the text to include examples of underlying causes of vaccine-related misconceptions, such as historical injustices, sociopolitical mistrust, and fears of Western exploitation. These additions help to contextualise the persistence of vaccine hesitancy and emphasise the importance of culturally sensitive, community-led strategies. The revised text now reads: "Efforts to combat vaccine hesitancy must be sustained and embedded within long-term public health strategies, rather than implemented reactively during outbreaks. Persistent myths and misconceptions—often stemming from historical injustices, sociopolitical marginalisation, or fears of Western exploitation and medical malevolence—require culturally sensitive, community-led approaches to be effectively addressed. This is particularly relevant in contexts where mistrust in health systems continues to shape public perceptions of vaccination campaigns. Building public trust demands continuous engagement through transparent communication, collaboration with local leaders, and the integration of behavioural and social sciences into public health programming. Improving community literacy levels in African communities was also posited as an avenue to improve acceptance of public health interventions such as vaccines "

Line 479-485: You can add some strength of the review study to make a balance.
Reply: Thank you for this key observation. The paragraph on limitations has bee rephrased to more clearly highlight the strengths and novelty of the work. It has been rephrased as follows: "While the study is primarily based on a scoping review methodology, limiting the application of statistical tests and resulting in largely descriptive recommendations, there are several notable strengths. The review synthesises a broad and complex body of evidence on cholera control in Zambia, offering a consolidated narrative that clearly outlines the key challenges and persistent gaps in the country's elimination efforts. By mapping these barriers against existing interventions, the study provides practical insights that can inform more targeted and strategic planning for the next iteration of the National Cholera Control Plan."

Line 541: Now or new?
Reply: Thank you for pointing this out. The phrase has been deleted to improve clarity and avoid ambiguity

Reviewer 2

Reviewer #2: The Methods need to be clarified, coherence with the research questions defined in introduction and the structure of the Results section improved, and further details provided

on how data were analysed or synthesised. The PRISMA check-list (for scoping or systematic review) is a useful guide to structure the manuscript - it is mentioned in Results (L. 122) but not adhered to.

Reply: Thank you for this valuable feedback. We have clarified the Methods section to ensure alignment with the research questions by specifying that selected papers were analysed according to thematic pillars and assessed for their aims, study design, population, geographic focus, identified risk factors, and proposed mitigative strategies. Additionally, we have improved the structure of the Results section for greater coherence and included the completed PRISMA-ScR checklist in the annexes to guide transparency and reporting.

The terms "scoping review" and "narrative review" are used interchangeably in the manuscript to refer to the work. However, the research questions defined at L. 97-99 require synthesising evidence on (i) cholera in Zambia and (ii) cholera control strategies, and they would be best addressed through a systematic review (see Munn et al., BMC Medical Research Methodology, 2018).

Reply: Thank you for the comment. We have clarified that a scoping review was intentionally chosen over a systematic review, as our primary objective was to comprehensively map the breadth of existing literature, the range of subtopics, and in order to identify emerging themes related to cholera control in Zambia. This approach was better suited to our broad research questions and the need to capture diverse evidence and contextual insights to inform national strategies.

L. 112: the search terms included "Zambia" and articles not related to Zambia were excluded. This makes the scope of the review very specific to the Zambian context and prevents identification of relevant evidence from similar settings to address the research question.

Reply: Thank you. We intentionally limited the scope to Zambia because our goal was to identify context-specific strategies and implementation challenges unique to the country, which are often not adequately addressed in broader regional literature. While evidence from similar settings is valuable, our focus was on generating tailored insights to inform national planning—complementary reviews can certainly explore cross-country comparisons. We do respectfully disagree though that a focus on the Zambian situation equals lack of relevance to other settings per se. We do believe extrapolation and critical comparison with other settings is possible and of interest to others, but beyond the chosen scope of our work presented here.

L. 109 / 118: what is the point of mentioning Embase if this database could not be accessed? Consider moving to discussion / limitations rather than keeping this in Methods and Results.

Reply: Thank you for this helpful suggestion. We have updated the respective text portion to clarify that while initial access to Embase was limited, this challenge was subsequently resolved, and relevant articles identified through Embase were ultimately included in the final analysis. We agree that discussing such access issues is more appropriate in the limitations section and have made that adjustment accordingly.

Results:

Reviewer #2: The Results section summarises findings from studies included in the review, but does not include any assessment of the strength of evidence. This is related to my comment above; a systematic review with a risk-of-bias assessment would be a more appropriate approach to synthesise evidence and formulate recommendations on that basis.

Reply: Thank you for this important observation. As this was a scoping review, we did not assess the strength or quality of the evidence, in line with the methodological guidance for scoping reviews, which aim to map the breadth of available literature rather than evaluate its rigour. Given the broad and exploratory nature of our research questions, focused on understanding the landscape of cholera-related research in Zambia and identifying emerging strategies, a systematic review with a risk-of-bias assessment was not appropriate for our objectives.

L. 111: what was done with the 11,300 records from Google Scholar and why are they not reflected in Fig 1? It is difficult to reconcile the manuscript text with Fig 1, as several numbers do not match.

Reply: Thank you for pointing this out. The figure has been updated to accurately reflect the number of records retrieved from Google Scholar (13 and not 11300), based on the search terms using ‘cholera’ AND ‘Zambia’, limited to January 2013 to June 2024. The corresponding numbers throughout the manuscript have been revised for consistency. We appreciate your attention to detail, which helped improve the clarity and accuracy of the review process presented.

L. 123-126: this belongs to Methods.

Reply: Thank you for spotting this. We agree that this information belongs in the Methods section, and we have ensured the updated numbers and description of the Google Scholar search are now clearly reflected there, along with the revised figure.

L. 147-148: only two papers in Table 1 appear to include more than one year of cholera surveillance and support this statement, and there is likely an issue with the reference number (Table 1 vs. text).

Reply: Thank you for pointing this out. We have reviewed the references and corrected the numbering discrepancy between the text and Table 1. Additionally, we have revised the table to clarify that only three studies provided multi-year surveillance data. The other sources contributed contextual information and were placed in appropriate sections within the text and removed from the table for the avoidance of confusion.

L. 148-152: this sentence is unclear, especially "...the proportion of clinically suspected cholera cases and not all confirmed by culture..." - are many cases confirmed by culture outside clinical settings?

Reply: Thank you for this observation. We agree that the original phrasing lacked clarity and have revised the sentence to better convey the intended meaning. The updated text now reads: 'This may be a limitation, as cholera cases are only reported in the IDSR system when an outbreak is confirmed by culture. Consequently, in endemic areas where suspected cases are not routinely subjected to laboratory confirmation, the true incidence and overall disease burden may be significantly underestimated, particularly outside of outbreak season.'

L. 187, 189: I believe 'Vibrio' is used instead of 'vibriones/vibrioni' in Englishs - to be checked.

Reply: Thank you for the correction. We have reviewed the terminology and replaced 'vibriones/vibrioni' with the correct term 'Vibrio in the revised manuscript'.

L. 213-217: how many studies is this estimate of 45% facility vs. 55% community deaths based on, and which time period does it refer to? Include references.

Reply: Thank you for this important observation. The estimate of 45% facility-based deaths versus 55% community deaths is based on two studies included in the review, which reported mortality patterns during recent cholera outbreaks in Zambia. We have clarified this in the revised text and added the appropriate references to reflect the time periods and sources of these data. The revised text reads "There was a slightly higher proportion of patients documented to have died before arrival at the treatment facilities (i.e. at home or community deaths) versus in the facility in the 2024 outbreak in Lusaka (60% community deaths) [6], proportionately more than the community deaths reported in the 2018 outbreak (45%)."

L. 223: "antimicrobial resistance" is vague - does it have any implications for patient management / treatment?

Reply: Thank you for this observation. We have clarified the statement to explicitly note that antimicrobial resistance (AMR) has direct implications for patient management in cholera treatment settings. Specifically, AMR can compromise the effectiveness of antibiotics used for managing severe cases, potentially leading to prolonged illness, increased risk of complications, and greater strain on clinical resources. We have updated the manuscript to reflect these implications and provide context for why surveillance of resistance patterns is important in outbreak settings.

L. 235: don't you mean that reported administrative coverage is higher than actual coverage?

Reply: Thank you for the clarification. You are correct—the sentence has been revised to reflect that reported administrative coverage may overestimate actual vaccination coverage.

Conclusions:

Reviewer #2: It is not entirely clear how the Discussion and Conclusions are supported by the Results. For instance:
- The statement at L. 314-316 may be sensible but how it is supported by evidence (from the Results section) on the effectiveness of decentralised surveillance and case-area targeted interventions

Reply: Thank you for this important observation. We have revised the relevant section of the Discussion to better align with the evidence presented in the Results. Specifically, we now refer to findings from Lusaka, where population movement contributed to the re-introduction of cholera across peri-urban wards [29], to support the argument for decentralised, community-centric interventions. We have clarified that such risk reinforces the need for localised surveillance and response systems, such as Case-Area Targeted Interventions (CATI), which have shown effectiveness in other high-risk settings including Uganda, the DRC, and Burundi [30]. The revised paragraph provides a clearer linkage between the study findings and the rationale for recommending decentralised approaches

- Fig 2 shows that there are very few publications on Community Engagement - but this is not reflected upon in Discussion (for identifying future research needs)

Reply: Thank you for this helpful observation. We agree that the limited number of publications on community engagement in cholera control is a significant finding. We have now included this point in the Discussion under the section on future research priorities. Specifically, we

highlight the need for more implementation research to identify effective, scalable models for community engagement in cholera surveillance, vaccination uptake, and WASH interventions. Strengthening the evidence base on what works in community engagement, particularly in informal settlements and mobile populations, is essential for achieving sustainable cholera control in Zambia and similar settings

Editorial and Data Presentation Modifications?

Use this section for editorial suggestions as well as relatively minor modifications of existing data that would enhance clarity. If the only modifications needed are minor and/or editorial, you may wish to recommend “Minor Revision” or “Accept”.

Summary and General Comments:

Reviewer #2: This review is relevant in the context of cholera resurgence in Zambia and across the continent. However, a systematic review including an assessment of the strength of evidence related to the effectiveness of different intervention strategies would more appropriate to address the research questions and inform the development of a sound strategy for cholera elimination. The inclusion from other settings outside Zambia should also be considered to strengthen the evidence synthesis and inform the prioritisation of interventions. I have provided more detailed comments under the respective manuscript sections.

<u>Reply:</u> We sincerely appreciate the reviewer’s opinion, but for a variety of reasons (alluded to above). However, we decided that for the research question posed, a scoping review format was deemed most appropriate to map the scope, characteristics, and implementation challenges of cholera control interventions in Zambia. The decision to focus on Zambia was deliberate, reflecting both the pressing national resurgence of cholera and the expertise of the study team. However, the approach chosen does neither pre-empt similar undertakings for other countries, nor does it mean that a wider regional review would not be possible. the methodology can serve as a foundation for similar reviews in other countries or as part of a broader regional synthesis. We have clarified this rationale in the manuscript.
