# Supplementary material for: Navigating the cholera elimination roadmap in Zambia – A scoping review (2013–2024)
Source: PLoS Negl Trop Dis. 2025 Jun 23;19(6):e0012422. doi: 10.1371/journal.pntd.0012422 (PMC12208409; doi:10.1371/journal.pntd.0012422)
Supplement: Attachment — Submitted filename: Review.Comments.PNTD-D-24-01118.docx [file pntd.0012422.s002.docx]

Dear Authors

This is a very well-written manuscript which will give information to the GTFCC as well as other partners involving the ending cholera 2030, global roadmap.

I have a few comments which are as follows:

**Abstract:**

Well structured.

Line 27: Kindly add types of study

Line 28-30: Adding how the risk factors affect the Zambia’s cholera elimination program would be beneficial.

Line 32: Indicate whether high-risk areas or the entire country will host climate-resilient WASH infrastructures.

Line 43: Keyword GTFCC – Use full form

**Introduction:**

The introduction demonstrates well about the context.

Line 62-63: Need to add cholera epidemiological data especially for sub-Saharan Africa as well as Zambia

Line 64-66: Add the measures already implemented in Zambia to address the nation’s effort before the 2024 outbreak for more clarity.

Line 72-74: Mentioning Health care system strengthening with Community engagement will add more depth. Also use only ‘WASH’ in the last for better clarity.

Line 92-93: How one heath approach will help Zambia cholera control approach. You can add some on this.

**Method:**

Method is clear and sound.

Line 105-106: Need to add some examples of research gap.

**Results:**

Line 119: Google scholar search found 11300 articles which is too high. How could you find the relevant one? Please explain and add in the section.

Line 122-123: A brief explanation of the impact of PRISMA rules on data extraction quality is needed for Unfamiliar readers.

Line 141-152: Epidemiology trends over time can be clarified by additional epidemic case numbers, regions, and age groups of affected individuals.

Line 221-223: Details and extent of AMR is needed for better description

Line 276-77: How el nino make impact on cholera outbreak? Kindly describe.

**Discussion:**

Well written.

Line 310-311: Could you please add the period or time

Line 331: Add some detailed information about mixed results of microbiome.

Line 332-334: You can add some causes of misconception.

Line 365: You can add some strength of the review study to make a balance.

Line 391: Now or new?
